# Pattern of QTc prolongation in Methadone Maintenance Therapy (MMT) subjects receiving different methadone dosages: A prospective cohort study

**DOI:** 10.12669/pjms.295.3395

**Published:** 2013

**Authors:** Nasir Mohamad, Muhammad Irfan Abdul Jalal, Azlie Hassan, Muslih Abdulkarim Ibrahim, Roslanuddin Salehuddin, Nor Hidayah Abu Bakar

**Affiliations:** 1 Nasir Mohamad, MD, MMed, Department of Emergency Medicine, School of Medical Sciences, USM, 16150, Kubang Kerian, Kelantan, Malaysia.; 2 Muhammad Irfan Abdul Jalal, MBChB, MSc (Medical Statistics), INFORMM, USM, 16150, Kubang Kerian, Kelantan, Malaysia.; 3 Azlie Hassan, MD, MMed, Department of Emergency Medicine, School of Medical Sciences, USM, 16150, Kubang Kerian, Kelantan, Malaysia.; 4 Muslih Abdulkarim Ibrahim, B.Pharm, Msc (Pharmacy), INFORMM, USM, 16150, Kubang Kerian, Kelantan, Malaysia.; 5 Roslanuddin Salehuddin, MD, MMed, Department of Emergency Medicine, School of Medical Sciences, USM, 16150, Kubang Kerian, Kelantan, Malaysia.; 6 Nor Hidayah Abu Bakar, MD, MMed, Department of Pathology, Hospital Sultanah Zainab 2, 15860, Kota Bharu, Kelantan, Malaysia.

**Keywords:** Methadone, QTc, High Dose, Low Dose

## Abstract

***Objectives:*** This study aimed to compare the QTc interval between low and high dose methadone groups and evaluate the pattern of QTc variation.

***Methods:*** This is a prospective cohort study conducted from December 2010 till August 2011 at Malaysian University of Science’s Hospital. Forty six subjects, grouped in high dose (>80mg) and low dose (<80mg) oral methadone, were followed-up at 4-weekly for QTc measurements. Relevant demographic and biochemical profiles were taken at intervals with concurrent QTc measurements.

***Results:*** No significant QTc differences between methadone dosage groups were found at Week 0 (434ms vs 444ms, *p* = 0.166) and week 8 (446.5ms vs 459ms, *p* = 0.076), but not at week 4(435ms vs 450ms, *p* = 0.029). However, there were significant associations between the groups with QT_c _prolongation at week 0 and 4 (OR 4.29(95% CI 1.01, 18.72) *p*=0.044 and OR 5.18 (95% CI 1.34, 20.06) *p* =0.013, respectively) but not at week 8 (OR 2.44 (95% CI 0.74, 8.01) *p*=0.139). On multivariate analysis, dose group was the sole significant factor for QT_c _prolongation for week 0 and 4 (*p *values 0.047 and 0.017, respectively), but not at week 8.

***Conclusion:*** High-dose methadone group is more likely to develop prolonged QTc than low-dose group. However, such effects were inconsistent and occurred even during chronic methadone therapy, mandating judicious QTc and serum methadone monitoring.

## INTRODUCTION

Methadone is a synthetic opioid, used mainly as an analgesic for chronic pain management and an opiate substitute in Methadone Maintenance Therapy (MMT). Methadone maintenance was first developed as a treatment for heroin addiction in mid-1960s and has been proven to be important in the reduction of crime rates and the spread of HIV among IVDUs.^1^^-^^3^ It possesses good bioavailability (70-90%), long half-life (mean 24 hours) which makes it an ideal option for opioid substitution therapy.^4^^,^^5^ With proper usage, methadone causes no sedation or intoxication and has few side effects which diminish over time.^6^

Many studies have demonstrated that methadone is associated with QTc prolongation.^7^^-^^10^ There is a clear relationship between dose and the magnitude of QTc prolongation for other drugs, for instance sotalol. However, this is less clear for methadone with conflicting evidence on the dose-dependent effects of methadone on cardiac repolarization. Martell et al showed that oral methadone results in QTc increases of 12.4 ms at 6 months, 10.7 ms at 12 months.^11^ However, Maremmani I et al (2005) did not find a significant correlation between methadone dose and QTc interval (r = +0.14).^12^

Similarly, even though it is generally agreed that the QTc prolongation is associated with relatively high doses of methadone, the exact dose at which this occurs is not known. To date, there is no available study on the effect of methadone dose on QT_c _interval in patients undergoing MMT in Malaysia. This study aimed to compare the QTc interval duration between low and high dose methadone groups over an eight-week period.

## METHODS

This is a prospective cohort study involving 46 subjects recruited from December 2010 until August 2011 among those receiving Methadone Maintenance Therapy (MMT) at Malaysian University of Science’s Hospital (HUSM). The inclusion criteria were 1) who fulfilled DSM IV criteria for opioid dependence (fulfilled minimum 3 out of 7 criteria: tolerance, withdrawal, taking larger amount of opioid or for longer duration than intended, persistent desire or unsuccessful effort to reduce opioid use, significant time spent in activities to obtain opioid, important social or occupational activities are abandoned or reduced, continued use of opioid despite knowing the problems experienced),2) consented to study participation. Subjects were excluded if; 1) had congenital long QT syndrome (LQTS) 2)co-medicated with drugs that altered serum methadone level (eg rifampicin, carbamezapine, ketoconazole, phenytoin etc) 2) polysubtance abusers, 3) age less than 18, 4) addicted to opioid for less than 2 years, 5) known hypersensitivity to methadone, 6) abnormal liver functions and 7) acute medical or psychiatric disorders. The purpose of excluding those being dependent on opioid for less than two years was to establish the chronic dependency status.

Relevant demographic and clinical data were acquired. An initial standard 12-lead ECG was performed for each eligible subject, at a paper speed of 250 mm/s and voltage of 10 mm/mV, using calibrated Welch Allyn CP 100 machine. Subjects with baseline QTc intervals greater than 500ms were excluded due to the possibility of fatal torsades de pointes. The QT measurements were corrected for heart rate (QTc) using Bazett’s formula = *QT* Interval / √ RR interval.^13^ QTc is considered prolonged if it exceeds >450ms for men and >470ms for women.^14^ Blood was taken for relevant biochemical profiles measurements.

All subjects were on chronic methadone treatment, thus achieving steady state methadone concentration. Subsequent follow-up ECGs were performed at 4 weekly for 8 weeks. The patients were dichotomized into those receiving methadone more than 80 mg or less than 80 mg. The 80 mg cut-off was chosen since this is the consensus for high dose and low dose methadone grouping as proposed by previous studies^15^^-^^17^ and our predetermined cut-off using Receiver Operating Curve (ROC) analysis^18^. All ECGs were performed 24 hours after last methadone dose with subjects remained supine for 5 minutes prior to ECG recording.

Sample size was calculated using Power and Sample Size freeware, Version 3.0.43 (2011 release, available at: http://biostat.mc.vanderbilt.edu/wiki/Main/PowerSampleSize ). According to Sundaram A et al^19^, the mean QTc in subjects receiving high dose methadone was 467.3 with a standard deviation (SD) of 42.6 while the mean QTc in low dose methadone group was 422.8 (SD±34.0). Therefore, fifteen subjects per group were needed to demonstrate the effect at alpha 0.05 and power 0.80. Informed consent was obtained from all participants and this study received an ethical approval from the University’s Ethical Committee (Ethical Approval Number: USMKK/PPP/JEPeM [221.3(5)].

***Statistical Analysis: ***Data analysis were performed using Statistical Package for Social Sciences (PASW) version 20.0 (IBM, New York) and STATA version 11 (StataCorp LP, Texas). The observed primary outcome was QTc values at each follow up visit with methadone dosage groups as the main factor. Serum calcium, potassium and magnesium, and co-medications known to prolong QTc interval (antiarrhythmics (amiodarone etc), antipsychotics (eg haloperidol), antidepressants (eg citalopram) antimicrobials (eg erythromycin) and others), comorbidities and age were treated as confounders. Continuous variables were described in mean (or median) and standard deviation (or interquartile range (IQR)) whilst categorical variables in frequency and percentage.

Independent-t or Mann-Whitney test was used to compare the QTc intervals between low and high dose methadone groups. Using bootstrap sampling procedures with bootstrap samples set at 1000, 95% confidence interval (CI) was computed. For categorical variables, chi square or Fisher’s exact test was used and Pearson’s correlation coefficient was calculated for methadone dose and QTc interval.

On multiple logistic regression, the outcome was dichotomized into two groups; prolonged or normal gender-specific QTc. The dose group was the main factor with effects on QTc adjusted for confounders.

## RESULTS

The total number of subjects included was 46, with 23 subjects receiving low dose methadone and 23 high dose methadone. The mean age of subjects was 35.9 years (SD 6.6 years, range 23-60). The full baseline patient characteristics are as in Table-I.

**Table-I:** Demographic and clinical profiles of subjects receiving methadone treatments (n=46).

*Variables*	*>80mg (freq / percentage)*	*<80mg (freq/percentage)*
Age	34.2 (5.7)^a^	37.6 (7.1)^a^
Gender		
Male	23 (50)	23(50)
Female	0 (0)	0 (0)
Alcohol		
Yes	0 (0)	0(0)
No	23(50)	23(50)
Other Drugs		
Nil	20(43.5)	21(45.7)
Antiretrovirals	1 (2.2)	3(6.5)
Antipsychotic	1 (2.2)	0(0.0)
HIV status		
Yes	1(2.2)	3(6.5)
No	22(47.8)	20(43.5)
Methadone dose	111.7(20.3)^b^	70.0 (10.8)^b^

^a^ Mean(standard deviation).

^b^ Mean methadone dose irrespective of groups was 90.9 (SD 26.6).

At Week-0, the median (IqR) QTc for low dose group was 434.00 (17.0) ms and the high dose group had a median QTc of 440.00 (40.0) ms, *p* = 0.166. At week-4, we found a significant QTc difference between the two methadone groups (435ms vs 450ms, *p* = 0.029). At week 8, the findings was similar to week-0 with no significant difference in mean QTc of low dose and high dose methadone group (446 vs 459, *p* = 0.076). The results are presented in Table-II.

**Table-II:** QTc interval (ms) between low dose and high dose methadone groups

	*Mean (SD)* ^+^ */ Median (IqR)*		*Mean difference (95% CI)*	*t stat* ^a^ */* *z stat* ^b^	*p value*
	*Low dose* *n = 23*	*High dose* *n = 23*			
Week 0	434(17)	440(40)	10.61(0.65,22.74)^c^	-1.39^b^	0.166
Week 4	435(21)	450(38)	12.13(2.94,21.29)^c^	-2.19^b^	0.029^*^
Week 8	446.48(21.94)^+^	459(24.67)^+^	12.52(-1.35, 26.40)	1.82^a^	0.076

^a ^Independent t test

^b ^Mann-Whitney test

^c ^Bias-corrected bootstrap 95% confidence interval

^*^ Statistically significant difference

+ Mean (SD)

**Fig.1 (a-c):**
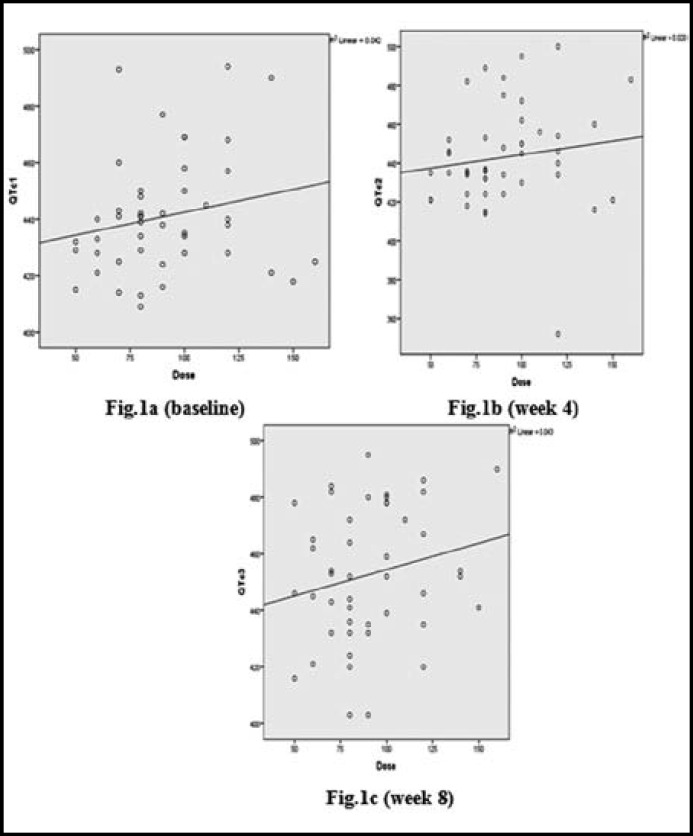
The scatter plot of QTc against the methadone dose from baseline to week 8. The R2 for each plots were 0.042, 0.020 and 0.043, respectively. The line represents best fit lines

However, there was significant associations between methadone dose group and QTc prolongation at week-0(OR 4.29 (95% CI 1.01, 18.72), *p* = 0.044) and week 4 (5.18 (95% CI 1.34, 20.016) *p=*0.013) but not at week 8 (OR 2.44 (95% CI 0.74, 8.01) *p*=0.139).  Besides that, there was no correlation between methadone dose (continuous figure) and QTc interval values for all three QTc measurements (methadone vs 1^st^ QTc Pearson’s r=0.205, p value=0.49, methadone vs 2^nd^ QTc Pearson’s r=0.141, p value=0.351 and methadone vs 3^rd^ QTc Pearson r= 0.207, p value=0.168), illustrated by scatter plots QTc vs Methadone dose (continuous) in Fig.1. On multiple logistic regression, dose group (>80 mg or <80mg) was the only significant factor for QTc prolongation at week 0 and week 4, but not at week 8 (Table-III).

**Table-III:** Factors associated with QTc prolongation using multiple logistic regression

*Periods*	*Variables*	*β(SE)* ^a^	*OR (95% CI)* ^b^	*LR stat(df)* ^c^	*p values*
0 week	Dose (>80 vs <80)	1.46(0.75)	4.29(1.01,18.72)	3.94(1)	0.047
4 weeks	Dose (80 vs <80)	1.65(0.69)	5.18(1.34,20.06)	5.67(1)	0.017
8 weeks	*				

^a^ Beta, SE= Standard error

^b^ OR= Odds ratio, CI = Confidence Interval

^c ^LR= Likelihood ratio, df=degree of freedom

* no factors associated with QT_c _prolongation

## DISCUSSION

We found that the percentage of prolonged QTc interval from week 0 to Week 8 ranges from 26.1% to 54.3%., similar to Perrin-Terrin A et al^20^. On the contrary, Maremmani I et al reported higher prevalence of age-and-sex-standardized QTc prolongation (83%)^12^ whilst Wedam F et al reported much lower prevalence of QT_c _prolongation (<10%)^21^. However, both utilized different cut-offs for prolonged QTc (440 ms and 470 ms (males)/490ms (females), respectively).

In our study, significant QTc differences between methadone dose groups were only observed at week 0 and 4 which is explainable by QTc interval fluctuation during methadone maintenance therapy. This is supported by the prior studies where fluctuation of the QTc occurred during methadone therapy, resulting in different QTc at different time points.^22^^,^^23^ This may be explainable by the circadian variation of QTc.^24^ Nevertheless, since significant QTc differences are found at baseline and week 4, serum methadone monitoring should be judiciously performed at week 4 upon methadone dose stabilization.

 Besides, QTc fluctuation might also be attributed to racemic methadone mixture containing s-methadone which blocks *hERG*-coded voltage-gated iKr potassium channels more potently than r-methadone. Besides that, pharmacogenomic factors such as CYP2B6 polymorphism with 6*/6* genotype, whose prevalence was between 13% to 26% among different ethnicities in Malaysia, causes the slow metabolism of methadone resulting in methadone accumulation. This, coupled with *hERG* polymorphism, further accentuated the QT_c _prolongation in susceptible individuals thus contributing to QTc differences between methadone groups.^24^

This study, however, has several shortcomings. Firstly, it is a single-centre study which may question the representativeness of the sample and its generalizability to the entire MMT population. Besides that, there is a lack of standardized definitions of prolonged QT_c_ that may hamper the outcome categorization. Apart from that, the study might also fail to exclude those with personal or significant family history of LQTS and other structural cardiac diseases (left ventricular hypertrophy, hypertrophic cardiomyopathy etc). It’s also highly recommendable to perform more frequent follow-up, especially at 2 weeks post baseline ECG measurement, since QT_c _prolongation might occur at earlier periods. Besides that, this study might be underpowered to significantly detect the QT_c_ difference for week 8. However, this is contrary to the fact that the number of study participants exceeded the apriori sample size calculation (post-hoc power=0.938) with 95% CIs estimated using bootstrapping technique.

## CONCLUSION

We concluded that subjects who received daily methadone dose of greater than 80 mg are more likely to develop prolonged QTc interval compared to subjects receiving less than 80 mg at baseline and week 4. However, the effects were inconsistent, suggesting a complex and fluctuating nature of methadone-induced QTc interval prolongation. Therefore, this merits for frequent QTc and serum methadone monitoring especially in high dose chronic methadone users.

## Notes

***Author’s contribution:*** NM is the study’s principal investigator, MIAJ responsible for statistical analysis, final drafting and intellectual content, AH and RS for data collection and early drafting, MAI and NHAB as secondary intellectual contributors. All authors have read and approved the manuscript.

## References

[1] Viswanath B, Chand P, Benegal V, Murthy P (2012). Agonist treatment in opioid use: advances and controversy. Asian J Psychiatr.

[2] Schwartz RP, Jaffe JH, O’Grady KE, Kinlock TW, Gordon MS, Kelly SM et al (2009). Interim methadone treatment: Impacts on arrest. Drug Alcohol Depend.

[3] Lobmann R, Verthein U (2009). Explaining the effectiveness of heroin-assisted treatment on crime reductions. Law Hum Behav.

[4] Leppert W (2011). Pain management in patients with cancer: Focus on Opioid Analgesics. Curr Pain Headache Rep.

[5] Portenoy RK (2011). Pain 3: Treatment of cancer pain. Lancet.

[6] Modesto-Lowe V, Brooks D, Petry N (2010). Methadone deaths: risk factors in pain and addicted populations. J Gen Intern Med.

[7] Krantz MJ, Kutinsky IB, Robertson AD, Mehler PS (2003). Dose-related effects of methadone on QT prolongation in a series of patients with torsade de pointes. Pharmacotherapy.

[8] Sala M, Anguera I, Cervantes M (2003). Torsade de pointes due to methadone. Ann Intern Med.

[9] Walker P, Kelin K, Kasza L (2003). High dose methadone and ventricular arrhythmias; a report of three cases. Pain.

[10] Sanju G, Moreira K, Fapuhunda M (2008). Methadone and the heart: What a clinician needs to know. Curr Drug Abuse Rev.

[11] Martell BA, Arnsten, JH, Ray B (2003). The impact of methadone induction on cardiac conduction in opiate users. Ann Intern Med.

[12] Maremmani I, Pacini M, Cesaroni C, Lovrecic M, Perugi G, Tagliamonte A (2005). QTc interval prolongation in patients on long-term methadone maintenance therapy. Eur Addict Res.

[13] Bazett HC (1920). An analysis of the time-relations of electrocardiograms. Heart.

[14] Mayet S, Gossop M, Lintzeris M, Markides V, Strang J (2011). Methadone maintenance, QTc and torsade de pointes: Who needs anelectrocardiogram and what is the prevalence of QTc prolongation?. Drug Alcohol Rev.

[15] Bracken BK, Trksak GH, Penetar DM, Tartarini WL, Maywalt MA, Dorsey CM, Lukas SE (2012). Response inhibition and psychomotor speed during methadone maintenance: impact of treatment, duration, dose and sleep deprivation. Drug Alcohol Depend.

[16] Lambers FAE, Stolte IG, van de Berg CHSB, Coutinho RA, Prins M (2011). Harm reduction intensity-its role in HAART adherence among drug users in Amsterdam. International Journal of Drug Policy.

[17] Farrell M, Wodak A, Gowing L (2012). Maintenance drugs to treat opioid dependence. BMJ.

[18] Mohamad N, Abu Bakar NH, Musa N, Talib N, I Rusli (Harm Reduction Journal ). Better retention of Malaysian opiate dependents treated with high dose methadone in methadone maintenance therapy.

[19] Sundaram A, Nafissi A, Schweitzer P, Dvorkin E, Homel P, Fredman D (2011). Effect of oral methadone on the QTc interval in patients with increased risk for QTc prolongation. J Pain.

[20] Perrin-Terrin A, Pathak A, Lapeyre-Mestre M (2010). QT interval prolongation: prevalence, risk factors and pharmacovigilance data among methadone-treated patients in France. Fundam Clin Pharmacol.

[21] Wedam FE, Bigelow GE, Johnson RE, Nuzzo PA, Haigney MCP (2007). QT-Interval Effects of Methadone, Levomethadyl, and Buprenorphine in a Randomized Trial. Arch Intern Med.

[22] Fredheim OM, Borchgrevink PC, Hegreanaes L, Kaasa S, Dale O, Klepstad P (2006). Opioid switching from morphine to methadone causes a minor but not clinically significant increase in QTc time: a prospective 9-month follow-up study. J Pain Symptom Manage.

[23] Rademacher S, Dietz R, Haverkamp W (2005). QT prolongation and syncope with methadone, doxepin and a ß-blocker. Ann Pharmacother.

[24] Musa N, Zulkafli MI, Talib N, Mohamad N, Fauzi H, Ismail R (2012). Haplotypes frequencies of CYP2B6 in Malaysia. J Postgrad Med.

